# Trends and Age-Period-Cohort Effects of Fertility Rate: Analysis of 26,224 Married Women in Taiwan

**DOI:** 10.3390/ijerph16244952

**Published:** 2019-12-06

**Authors:** I-Shiang Tzeng, Kuo-Hu Chen, Yungling L. Lee, Wen-Shan Yang

**Affiliations:** 1 Department of Research, Taipei Tzu Chi Hospital, Buddhist Tzu Chi Medical Foundation, New Taipei City 23142, Taiwan; istzeng@gmail.com; 2 Department of Statistic, National Taipei University, Taipei 10478, Taiwan; 3 Department of Applied Mathematics; Department of Exercise and Health Promotion, Chinese Culture University, Taipei 11114, Taiwan; 4 Department of Obstetrics and Gynaecology, Taipei Tzu Chi Hospital, Buddhist Tzu Chi Medical Foundation, New Taipei City 23142, Taiwan; alexgfctw@yahoo.com.tw; 5 Institute of Epidemiology and Preventive Health, College of Public Health, National Taiwan University, Taipei 10055, Taiwan; 6 Institute of Biomedical Sciences, Academia Sinica, Taipei 11529, Taiwan; 7 Institute of Sociology, Academia Sinica, Taipei 11529, Taiwan

**Keywords:** fertility, age-specific fertility rate, women, cohort effects

## Abstract

Taiwan and a few Asian societies have had among the lowest fertility rates in the world for the past decade. Understanding the reasons behind the low fertility and designing policies accordingly to improve fertility has been a priority of governments in the region. It what follows we examine the low fertility rate in Taiwan by studying the trend of actual fertility rate and desired fertility rate in Taiwan using an age-period-cohort (APC) model. Using the Knowledge, Attitude, and Practice (KAP) of contraception survey data between 1973 and 2004, we applied APC analyses on the actual fertility rate and desired fertility rate of married women. We found that youngest cohorts (the mid-cohort year 1983) had 10% higher actual fertility and 15% higher desired fertility compared to those who were born in 1959–1965, respectively. Additionally, we attributed current lowest-low fertility (at or below 1.3) to late marriages. There is a lag between the actual and desired fertility rates in KAP survey due to tempo effect. Furthermore, the trends of the cohort effects of both fertility rates in KAP surveys are reversing in Taiwan. Consequently, increase total fertility rate (TFR) should encourage marriage among the marriageable population and reward married and childbearing households.

## 1. Introduction

Maintaining stable population development is an indispensable factor of socioeconomic growth. Lowest-low fertility may cause problems, such as a labor force shortages, the ageing of society, a lack of staff for the long-term care of elderly persons, and a demographic dividend reduction [1,2,3]. In Taiwan, fertility has declined significantly since the late 1950s. However, in 1965, the beginning of the family planning program accelerated this demographic trend [4]. Although the control of the population growth with the goal of increasing the robustness of the economic system caused the total fertility rate (TFR, defined as births per women of childbearing age) to decline between 1951 and 1975, the rate increased in 1976. Additionally, the TFR has been lower than the replacement level since the mid-1980s. Furthermore, the TFR remained stagnant at 1.75 from 1991 to 1997 and declined further the next year. Despite a slight increase in the TFR in 2000 (Year of the Dragon), there has been a trend of decline in the TFR in the 21st century. Indeed, in 2010, the TFR in Taiwan was 0.89, which is the lowest in recorded history [5]. The TFR in Taiwan improved in 2011 (i.e., 1.06) and continued to increase in 2012 (i.e., 1.27), but it oscillated from 1.07 to 1.16 in 2013 and 2014.

Research on fertility and increasing fertility has focused on appropriated mathematical models and statistical analyses of fertility data [6]. Demographers and statisticians have utilized the results of these studies to promote fertility. In the past decades, age-period-cohort (APC) analysis has been a very popular method for studying social topics such as fertility [7]. The results of APC studies can provide policymakers with important evidence about the development of fertility policies. 

In a series of studies, the Taiwan Provincial Institute of Family Planning examined the knowledge, attitudes, and practices (KAP) of contraception use among married women [8,9]. In Taiwan, the desired numbers of children of women can provide useful clues for predicting their actual reproductive behaviors [10]. After the end of World War II, the global economy began to grow rapidly. The rapid advancement in medicine and technology has reduced the infant mortality rate, and then the baby boomers came out after the war. However, since the 1950s, experts began to discover that the population growth rate is too fast and may erode the economic growth. Attitudes towards fertility and contraception access have changed drastically during this time. Furthermore, awareness of the fertility attitudes of married women can provide insight into future fertility trends. The KAP contraception data also allow for the comprehension of the fertility rates among the married women of Taiwan between 1965 and 2004 [11].

This study aimed to provide a clear goal, measurements and an appropriate approach for the construct analyses of psychological factors related to childbearing in Taiwan. Based on the identification and analyses described below, surveys were used to collect data about contraception knowledge and use, pregnancy experiences, fertility, and family planning (i.e., denoted as KAP) to examine the relationship of psychological fertility with actual reproduction. We investigated the influence of birth cohort on the changes in psychological and actual fertilities after accounting of age and the survey period. Furthermore, this paper explored age-period-cohort analyses to provide useful clues about factors that specifically influence actual reproductive behaviors.

## 2. Materials and Methods 

### 2.1. Data Source

We used the KAP of contraception survey data collected by the Taiwan Provincial Institute of Family. The KAP survey in Taiwan has nine waves in total from the year 1965 to 2004, and collected data on contraception knowledge and behaviors, pregnancy experiences, fertility, family planning, family relations, and residence history [12]. Our study only used the last six waves, which are denoted as KAP IV, KAP V, KAP VI, KAP VII, KAP VIII, and KAP IX (we discarded KAPI-III due to the fact the time intervals of these surveys were too close). The investigative periods for the KAP surveys IV-IX were 1973, 1979, 1986, 1992, 1998, and 2004, respectively. The KAP surveys IV-IX have 26,224 participants, respectively. Note that we use KAP survey of married women in this study. 

We utilized two questions from the survey series to evaluate the actual and desired fertility rates. These two questions were, “How many live births have you had?” and “If you are currently married, how many children do you want to have in your life?” The formulas of actual and desired fertility are defined as follows:

$$\mathrm{The}\;\mathrm{actual}\;\mathrm{fertility}\;\mathrm{rate}=\frac{\mathrm{the}\;\mathrm{number}\;\mathrm{of}\;\mathrm{live}\;\mathrm{births}}{\mathrm{the}\;\mathrm{number}\;\mathrm{of}\;\mathrm{childbearing}\;\mathrm{women}\;\mathrm{who}\;\mathrm{aged}\;15\;\mathrm{to}\;49}\;\mathrm{The}\;\mathrm{desired}\;\mathrm{fertility}\;\mathrm{rate}=\frac{\mathrm{the}\;\mathrm{desired}\;\mathrm{number}\;\mathrm{of}\;\mathrm{children}}{\mathrm{the}\;\mathrm{number}\;\mathrm{of}\;\mathrm{childbearing}\;\mathrm{women}\;\mathrm{who}\;\mathrm{aged}\;15\;\mathrm{to}\;49}$$

These formulas were used for the total 26,224 individuals data that were cross-tabulated with four seven-year age groups (20–26, 27–33, 34–40, and 41–47), 5 seven-year time periods (1972–1978, 1979–1985, 1986–1992, 1993–1999, and 2000–2006), and eight birth cohorts (mid-cohort years: 1934, 1941, 1948, 1955, 1962, 1969, 1976, and 1983). Note that we discarded the childhood and adolescent groups (0–19 years old) from this analysis. Hence, we revised the formulas of actual and desired fertility, which listed as follows:

$$\mathrm{The}\;\mathrm{modified}\;\mathrm{actual}\;\mathrm{fertility}\;\mathrm{rate}=\frac{\mathrm{the}\;\mathrm{number}\;\mathrm{of}\;\mathrm{live}\;\mathrm{births}}{\mathrm{the}\;\mathrm{number}\;\mathrm{of}\;\mathrm{childbearing}\;\mathrm{women}\;\mathrm{who}\;\mathrm{aged}\;20\;\mathrm{to}\;49}\;\mathrm{The}\;\mathrm{modified}\;\mathrm{desired}\;\mathrm{fertility}\;\mathrm{rate}=\frac{\mathrm{the}\;\mathrm{desired}\;\mathrm{number}\;\mathrm{of}\;\mathrm{children}}{\mathrm{the}\;\mathrm{number}\;\mathrm{of}\;\mathrm{childbearing}\;\mathrm{women}\;\mathrm{who}\;\mathrm{aged}\;20\;\mathrm{to}\;49}$$

However, the actual and desired fertility rate data for those aged 41–47 in 1972–1978 period was not available, hence the mid-cohort year 1934 cohort was not available for the analysis. In additional, we also calculated the ratio of actual fertility to the desired fertility. If the ratio is larger than one, it means the women gave birth to more children than desired. Conversely, if the ratio is less than one, it means the women did not have the desired number of children. 

We used the mid-cohort year 1962 cohort as the reference cohort. We also select the youngest age-group (aged 20–26) as the reference age-group because it has the lowest actual and desired fertility rates compared to other age-groups. Given the mid-cohort year 1962 cohort and the 20–26 age-group being the reference groups, the reference group for period was the period 1979–1985 because the exact linear relation of the age, period, and cohort (cohort + age = period). 

Moreover, we analyzed the fertility rate data of the childbearing women in Taiwan (after discarding the childhood and adolescent groups) following a similar approach to compare the results of the birth cohort effects (Department of Statistics (DS) 1950–2009). In this component of the study, there were 5 five-year age groups ranging from 20 to 44 years old (20–24, 25–29, 30–34, 35–39, and 40–44), 8 five-year periods (1965–1969, 1970–1974, 1975–1979, 1980–1984, 1985–1989, 1990–1994, 1995–1999, and 2000–2004) and 12 cohorts (mid-cohort years: 1927, 1932, 1937, 1942, 1947, 1952, 1957, 1962, 1967, 1972, 1977, and 1982). The divergent widths of the effects of the KAP surveys and fertility statistics had no bearing on the purpose of this study.

### 2.2. Statistical Analysis

We used APC analyses used to measure the actual and desired fertility rate by parameters estimates (age, period, and cohort effects). In this study, we partitioned the marital fertility into the effects of age, period, and cohort to measure childbearing probability. Let the marital fertility of the *i*^th^ age group and the *j*^th^ period group be denoted by *λ_ij_*. The APC model is as follows:

log*λ_ij_ = μ + α_i_ + β_j_ + γ_k_, i = 1, 2,…,I*           *j = 1, 2,…,J*           *k = j – i + I* (1)

where the intercept term is represented by μ, the age effects by $\alpha_{i}$, the period effects by $\beta_{j}$, and the cohort effects by $\gamma_{k}$. The following constraints are used: $\sum_{i}\alpha_{i}=\sum_{j}\beta_{j}=\sum_{k}\gamma_{k}=0$. The free R software used to performed APC analysis to estimate the age, period and cohort effects.

### 2.3. Multiphase Method in Estimating Cohort Effects

In epidemiology, one popular interpretation of the relationship of age, period, and cohort variables is that age and period interact to create unique generational experiences. Greenberg et al. proposed the conceptualization of cohort effects as an interaction of age and period [13]; a cohort effect is viewed as a period effect that is differentially experienced through age-specific exposure to events or causes, like an interaction between age and period [14]. Although age, period and cohort still has an exact linear relation (cohort + age = period) in this conceptualization, exposures (i.e., predictors) are not intrinsic to birth cohorts.

We used the median polish analysis to estimate cohort effects under the above conceptualization [13,14]. The median polish method was developed to describe data in a two-way contingency table [15]. This median polish method requires no assumptions about the distribution or structure of the data in a two-way contingency table. In overall, row and column effects and the residuals are computed by the following median polish algorithm. In the first step, we compute the median for each row, keeping it as the row median and subtracting it from the values in the corresponding row. In the second step, similarly we compute the median for each column, still keeping it as the column median and subtracting it from the values in the corresponding column. Finally, the above two steps are repeated until the row and column medians do not change (or have very small changes). Consequently, it can be widely used for any data type in a table without any assumption, such as rates, logarithms of rates, proportions and counts. Selvin first applied the median polish method to APC analysis [16]. The main advantage of applying this method to APC analysis is that it removes the influence of age (i.e., row) and period (i.e., column) by subtracting the median from each row and column [15], and thus cohort may treat as an isolated factor from removing age and period effects. Another advantage of using the median polish method to APC analysis is that it allows missing data while the traditional estimation method does not. The median polish method removes median only in allowed missing column or raw data. The initial concept of the median polish method was introduced in our previous study [17]. 

In this study, we conceptualized age and period effects as additive increases to the actual and the desired fertility rates. That is, the related fertility rates (*R_ij_*) for the *i*-th age category and *j*-th year is modeled as:

(2) $$R_{ij}=\mu +\alpha_{i}+\beta_{j},$$

where μ is the underlying related fertility rates; $\alpha_{i}$ is the age effect; and $\beta_{j}$ is the period effect. In this model, both age and period independently influence $R_{ij}$. When a cohort effect exists, the influence of age is the same for all time periods and vice versa. It is not reasonable for Equation (1) to leave out cohort effect. Consequently, Selvin modified the original equation to:

(3) $$R_{ij}=\mu +\alpha_{i}+\beta_{j}+\gamma_{ij},$$

where $r_{ij}$ is a cohort effect, an interaction between age and period effects [16]. Theoretically, a cohort effect correlated with age and period effects. In this conceptual model, Selvin applied the median polish analysis to two-way contingency tables for age and period [16]. After several iterations, the cell values stabilize near zero leaving residual values that contain the cohort effect. The residual values measure the deviation of each cell from an additive model. Theoretically, the residuals should average to zero after removing the non-additive cohort effect. Moreover, Keyes and Li proposed the multiphase method, which extends Selvin’s work by separating the cohort effect and error terms [14]. There are three phases in our analysis. The first phase was the creation of graphs of the age variations across periods. These graphs provide information that aids the understanding of the presence of cohort effects. The second phase involved aforementioned median polish analysis to remove the additive effect of age and period by iteratively subtracting the median from each row and column. The third phase was the regression analysis. After the median polish, the residuals of the cohort category in a linear regression model were partitioned into systematic and non-systematic components. The systematic element considered the cohort effect, the remaining variance of the random error. Additionally, we compared each birth cohort category to the reference birth cohort to acquire each rate ratio that provides a relative estimate of the scale of that cohort effect. Theoretically, the rate of the reference birth cohort varies slightly after removing the influence of the cohort. To remove the cohort effect and determine the reference birth cohort, we multiplied factor *e^−(residuals)^* to the rate of each age group. If the ratio of log-additive rate without and with cohort effects is close to one, then it is determined as the referent birth cohort. In words, more probable cohort could be reference group with less residual between before and after removing cohort effects from rate. The 95% confidence intervals (CIs) of rate ratios were also calculated with the corresponding standard errors.

### 2.4. Curvature Change Points

We present change points in the curvatures of the actual and the desired fertility rate ratios (i.e., cohort effects) and these points obtained by fitting segmented regression model. The segmented analysis via free R software performed to estimate the change point and standard error for estimates of the cohort effects with the Davies’ test for segmented regression model [18]. The procedure can be conveniently fitted using the R package “segmented” [19]. The 95% CIs of the estimated change points are providing in this study.

## 3. Results

**Figure 1:**
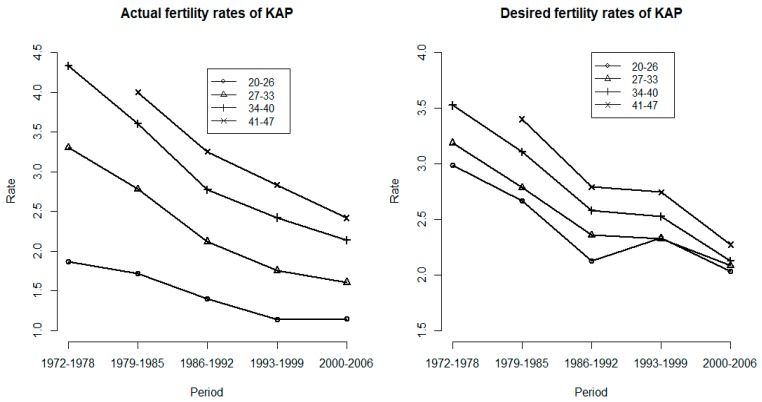
The actual and desired birth rates of age-specific groups by surveyed year, females, Taiwan, 1972 to 2006.

Figure 1 shows the actual and desired fertility rates by age group over time. In the left panel, the actual fertility rates for all age groups declined substantially at the previous sharper rate of decline till 1993. After 2000, the actual fertility rates increased slightly for those aged 20–26 but continued to decrease for all the other older age groups. In the right panel, the desired fertility rates by age group decreased at a relatively stable rate till 1986. Between the 1986-1992 and 1993–1999 periods, the desired fertility increased for those aged 20–26 and declined more slowly for all the other older age groups. After the 1993–1999 period, the desired fertility rates decreased for all age groups.

**Figure 2:**
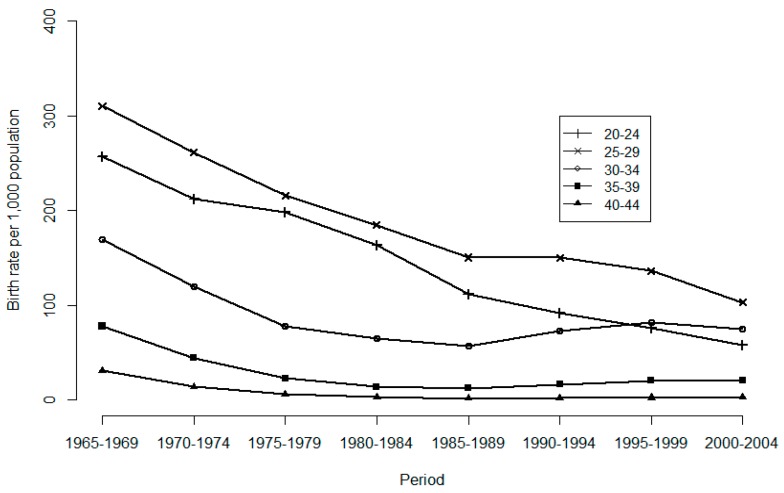
Age-specific birth rate per 1000 population by year, females, Taiwan, 1965 to 2004.

For age groups 27–33, 34–40, and 41–47, the actual fertility rates decreased faster than that in the 20–26 age-group. This non-parallel decreasing trend in actual fertility observed among these age groups indicated the existence of a cohort effect. Similar non-parallel trend for desired fertility were observed after the 1993–1999 period, suggesting a cohort effect on desired fertility as well. In addition, Figure 2 presents age-specific birth rates over time. After 1985, the birth rates increased for women aged 30–34 and 35–39, revealing a period effect. The birth rates also exhibited non-parallel trends across age groups, which indicated a cohort effect. We found that survey data of fertility and empirical data of birth rates are consistent in longitudinal trend.

Table 1 presents the estimated effects on the actual and desired fertility rates based on the progressive APC models. The APC model fit the data best given it has the smallest deviance (over the degrees of freedom) among all three models (Table 1).

**Figure 3:**
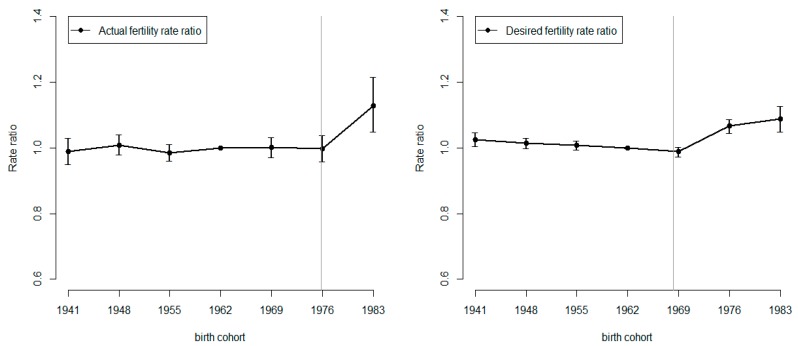
Graph of estimated rate ratios and 95% conference intervals for effect and change points of birth cohort on actual and desired fertility rates of KAP, Taiwan.

Figure 3 presents the cohort effects of the actual and actual fertility rates across the birth cohorts from the APC model. In the left panel (actual fertility rate) the cohort effect increases from 0.99 (mid-cohort year 1955 cohort) to 1.13 (the most recent birth cohort, the mid-cohort year 1983 cohort). Compared to the reference cohort, the actual fertility rate for the mid-cohort year 1983 cohort was approximately 15% higher. In the right panel (desired fertility rate), the increase was evenly distributed. Here, the cohort effect increased from 0.99 (mid-cohort year 1969 cohort) to 1.09 (mid-cohort year 1983 cohort), a 10% increase. Moreover, the change points obtained by segmented regression models were presented in Figure 3.

**Figure 4:**
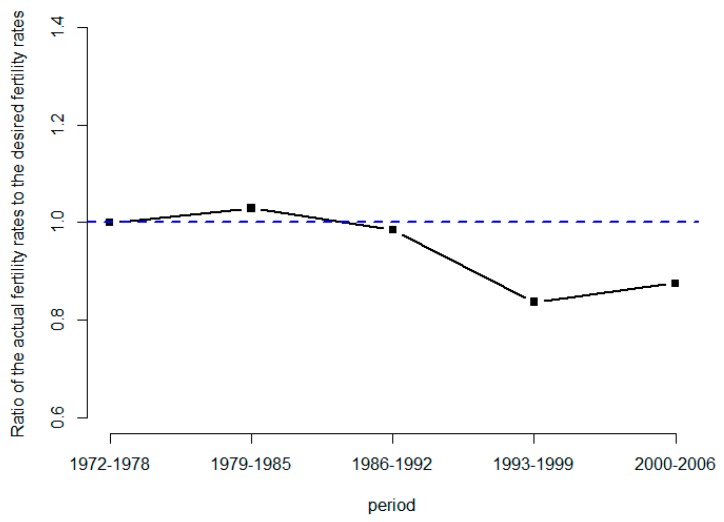
Graph of ratio of the actual fertility rates to the desired fertility rates of KAP, Taiwan.

Figure 4 shows the ratio of the actual fertility rates to the desired fertility rates over time. The ratio was roughly one during 1972–1978 and over one around the 1979–1985 period. The ratio dropped below one and remained less than one after 1985. The ratio first experienced a decline trend from 1985 to 1999 and then it increased during the 2000–2006 period. Note that the ratio of the actual fertility rates to the desired fertility rates may not be an appropriate measure for any women who are still in their childbearing years, because they might be planning more pregnancies. In the meantime, this ratio can provide a trend of difference between the actual fertility rates to the desired fertility rates for women who are past the age of childbearing. 

**Figure A1:**
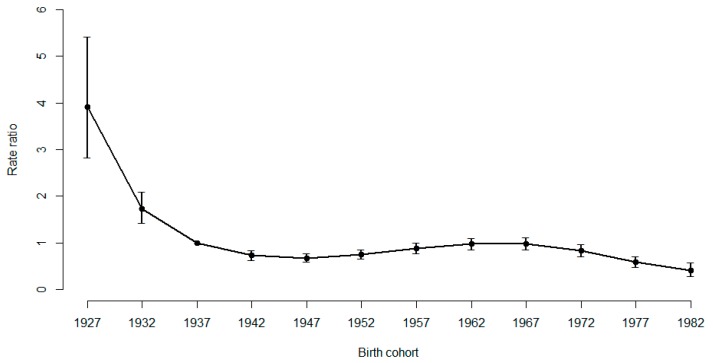
Graph of estimated rate ratios and 95% conference intervals for effect of birth cohort on fertility rates, Taiwan.

Among the birth cohorts, the women born near 1927 exhibited the highest rate ratio (Figure A1 in the Appendix was based on DS, 1950–2009). Note that age group age-group which aged 20–24 and cohort group with mid-cohort years 1937 were used as a reference group. Consequently, the rate ratio was 3.91 (95% CI: 2.83–5.41) for the 1927 birth cohort compared to reference birth cohort of 1937. There was also a dramatically decreasing trend for the earlier cohorts. Additionally, the rate ratios varied between the 1937 and 1982 cohorts. In this study, we limited our APC analysis of the median polish procedure to estimating the rate ratios and 95% CIs of the actual and desired fertility rates’ cohort effects.

## 4. Discussion

### 4.1. Fertility Trends

**Figure A2:**
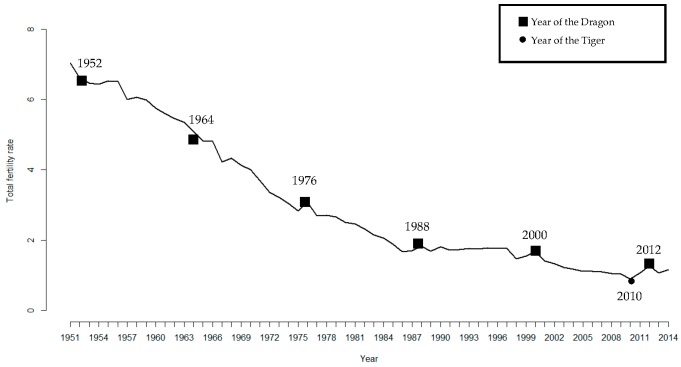
Graph of trends for total fertility rate, Taiwan, 1951 to 2014.

Based on direct observations of the long-term trends in TFR from 1951 to 1980 in Taiwan (see Figure A2 in the Appendix), there is no doubt that the trends revealed decreases in most of the years. Because the lowest Taiwan TFR ever recorded occurred in 2010, related predictions and studies have drawn public attention. One potential and traditional reason for these results in Asia is the effect of the Year of the Tiger (i.e., 2010) on fertility behavior. In the past, children born in Years of the Dragon (i.e., 1976 and 1988) have been more reproductive than those yields in Years of the Tiger. However, most recently, the Dragon year (i.e., 2012) failed to reproduce for the lower fertility rate, and fertility continued to decrease as usual during the Tiger year [20]. Furthermore, we observed that the latest higher reproduction, which occurred between 2010 and 2012, was substantially inflated compared with the previous cycles (1986–1988 and 1998–2000).

According to the 2012 age-specific national vital statistics, the 30–34 age group exhibited the highest fertility rate among all childbearing age groups (DS, 1950–2009). The 30–34 age group can retrospectively be placed in the 1978–1982 age group that overlaps with the most recent cohort of 1983 (birth cohort = 1980–1986) in actual KAP birth rates. In this study, the women born around 1983 exhibited the highest fertility rate of childbearing among the birth cohorts and provided the greatest contribution to increasing future fertility rates. The question then becomes, “Did the integration of these effects actually increase the higher reproduction between years?” In response, we attribute the most substantial effects to the premeditated fertility behavior that occurred in the Dragon year. Consistently, the TFR immediately dropped to 1.07 in 2013, representing a decrease of 0.2 compared with 2012.

The empirical findings of the current study indicating a behavior intention lag might lead to low fertility. Besides, women getting married later in life may also cause lowest-low fertility (i.e., TFR at or below 1.3) in Taiwan [21]. In the meantime, women getting married later can also cause female infertility. According to record from DS of the Ministry of Internal Affairs of Taiwan, the average age of first marriage with the men and women was 33 and 28 years old, and the median was 31 and 26.6 years old in 2004. But, the average age of first marriage was 28.2 for men and 22.1 for women, and the median was 26.8 and 22 years old in 1971. The first marriage age delayed with 3–6 years old among thirty years. Female fertility increases and then decreases after puberty, with advanced maternal age causing an increased risk of female infertility. Recently, the child mortality rate differences between Taiwan and Organization for Economic Cooperation and Development (OECD) countries had be investigated and found that child mortality rate, especially over 5 years of age, was higher than most OECD countries, including Japan and Korea [22]. The above factors would affect the number of actual and desired births, since people presumably desire a child.

### 4.2. Age Effects

Table 1 shows the age effects. The age effects are quite notable for both fertility rates. For actual fertility rates, the age effects increase from the younger age group, aged 27–33, to the oldest age group, aged 41–47. For desired fertility rates, the age effects increase from younger age group, but reverse slightly to the oldest age group. A previous study showed that fertility intension may correlate with women’s age [23]. Notable the desired behaviour of the 20–26 group in 1993–1999 in Figure 1. We examined that women’s economic status may be improved as education has expanded rapidly and lead to change in desired behaviour of the 20–26 group in 1993–1999.

### 4.3. Tempo Effects

If the age-specific fertility rates of the population varied over time, then TFR may not be a precisely measure of completed fertility (CFR, total number of live births in the childbearing years). Then, there is a stable effect which may exist on age-specific fertility rates, defined as a tempo effect. Additionally, the below-replacement level of fertility in Taiwan since the mid-1980s has primarily been attributable to the tempo effects (i.e., period effects) of postponing childbearing, which also reduced the TFR of the period [24]. In this study, the actual birth rates show a decrease substantially compared with the desired birth rates in the recent period (2000–2006). Note that the ratio of the actual fertility rates to the desired fertility rates was 0.88 in the period 2000–2006 (Figure 4). It is also important to note that the tempo effect caused a gap between the actual and desired rates in KAP survey IX. According to the study [25], KAP summarized as four models based on mutual relationships. Attitude and practice of procreation may mutually have influence on to each other.

### 4.4. Causes of the Tempo Effects

Indeed, there are many uncertainties regarding the tempo of childbearing. Much of the existing literature has demonstrated that increased human capital expenditures per child are associated with lowest-low fertility [1,26,27]. The implication of this finding is that a portion of the first demographic dividend among the demographic transition invested in human capital but reinforces the economic benefits of fertility declines.

### 4.5. Cohort Effects

However, the trends of the cohort effects of desired and actual birth rates in KAP surveys are reversing in Taiwan. Notably, the attitudes toward childbearing have changed among the more recent cohorts of married women. To be more specific, regarding the KAPs of recent birth cohorts, the cohort effects of actual births have increased after those of desired births. That is, there is a period lag between the recent cohort effects of desired and live birth rates and we can interpret it as behavior intention lag. The behavior intention lag also observed between the change points of cohort effect of actual birth rate in generation born from 1973 to 1979 (estimate:1974.98; 95% CI: 1967.79–1982.17) and that of desired birth rate in generation born from 1966 to 1972 (estimate: 1968.26; 95% CI: 1962.56–1973.96). Additionally, the cohort effect of the youngest birth cohort is lower than previously and consists of a continuous decline in the fertility rates of women of childbearing age in Taiwan. Specifically, the cohort effect of the actual birth rate is highest among the most recent birth cohort of 1980–1986 in the KAP, suggesting that the younger birth cohort of married women would have more births than that of the older cohort living in the same period. Furthermore, the last 6 stages of the KAP survey series reveal that the majority of women’s live birth rates are greater than the desired birth rates of the earlier period.

### 4.6. Limitations

The limitations of our study included the need for the median polish phase to achieve identification problem of the effects of age, period, and cohort. Unlike other approaches for modeling cohort effects [17,28], the median polish approach defines the cohort effect as a multiplicative interaction between age and period. That is, the cohort effect defined as a second order effect without a linear component. The linear components are conventionally the additive age and period effects. The cohort effect has no independence from the age and period effects; thus, the multiphase method provides a quantitative estimate of the second-order effect, which treated as an age-by-period interaction in the interpretation of the analysis results. In the real, some determinants about knowledge or attitudes may relate to fertility. Those determinants may treat as confounding factor while investigating association between fertility and influential predictors. However, none of the possible fertility-confounding factor accounted for in our study such as levels of education and occupation. The assessment of the survey sample has no bearing without considering this information in our study.

## 5. Conclusions

The cohort effects of desired fertility rates may ahead a period lag than that of actual fertility rates in KAP survey due to tempo effect. Although the trends of the cohort effects of both fertility rates in KAP surveys are reversing in Taiwan, we expect the total fertility will further to decline in the near future as evidenced in the results of the present study. Consequently, encourage the marriageable population to get married and reward childbearing married households may promote as fertility policies. Besides, simultaneously boost fertility intension and improve child mortality rate may also have a certain influence on fertility rates. Although these policies alone cannot fully restore the fertility rates to previous levels and ensure appropriate replacement, such policies should be a priority to retard the trend of declining fertility in Taiwan by encouraging, rewarding, and supporting childbearing.

## Figures and Tables

**Table 1:** Estimated effects and deviance based on progressive models on actual and desired fertility rates in Taiwan, 1972 to 2006.

Model	Actual Fertility			Desired Fertility		
	A	AP	APC	A	AP	APC
Age (*α_i_*)						
20–26	REF	REF	REF	REF	REF	REF
27–33	−0.54	−0.71	−0.67	0.21	0.10	0.10
34–40	0.64	0.50	0.50	0.81	0.71	0.70
41–47	0.81	0.65	0.67	0.79	0.69	0.68
Period (*β_j_*)						
1972–1978		0.72	0.75		0.64	0.64
1979–1985		REF	REF		REF	REF
1986–1992		1.05	1.07		1.03	1.03
1993–1999		−0.86	−0.86		−0.77	−0.79
2000–2006		−0.71	−0.71		−0.70	−0.69
Mid-Year of birth (*γ_k_*)						
1934			NA			NA
1941			0.99			1.03
1948			1.01			1.01
1955			0.99			1.01
1962			REF			REF
1969			1.00			0.99
1976			1.00			1.07
1983			1.13			1.09
df	16	12	5	16	12	5
Deviance	1600.778	27.938	21.587	740.663	10.944	4.371
Deviance/df	100.049	2.328	4.317	46.291	0.912	0.874
*p*-Value	<0.0001	<0.0001	<0.0001	<0.0001	<0.0001	<0.0001

Note: A = age model; AP = age-period model; APC = age-period-cohort model; NA = non-available; REF = reference.
